# Unilateral Nevoid Hyperkeratosis of the Nipple and Areola: A Case Report of a Rare Entity

**DOI:** 10.7759/cureus.98905

**Published:** 2025-12-10

**Authors:** Fatema Shamsaldeen, Alsadat Mosbeh, Abeer Albazali

**Affiliations:** 1 Internal Medicine, Farwaniya Hospital, Sabah Al Nasser, KWT; 2 Dermatology/Dermatopathology, Faculty of Medicine, Al-Azhar University, Cairo, EGY; 3 Dermatology, Farwaniya Hospital, Sabah Al Nasser, KWT

**Keywords:** benign, epidermal proliferation, hyperkeratotic nipple lesion, nevoid hyperkeratosis of nipple, nipple lesion

## Abstract

Nevoid hyperkeratosis of the nipple and areola (NHNA) is a rare benign skin disorder that presents as hyperkeratotic, verrucous plaques involving one or both nipples and areola. Although the lesions are asymptomatic, they can raise cosmetic concerns and may clinically mimic serious conditions like Paget’s disease. Histopathology examination is essential for diagnosis and for ruling out malignant conditions. We report a 72-year-old Kuwaiti male with unilateral verrucous plaques persistent over the nipple and areola for six months. Physical examination and mammography revealed no abnormalities, and biopsy showed marked hyperkeratosis, acanthosis, papillomatosis and superficial lymphocytic infiltrate without atypia, consistent with NHNA. The patient was treated with topical corticosteroids with a good response. This case highlights a unilateral male presentation of NHNA, emphasizing the importance of recognizing this benign entity from other malignant diseases like Paget’s disease. Biopsy is essential for accurate diagnosis, ensuring malignancies are excluded, and appropriate therapy is initiated.

## Introduction

Nevoid hyperkeratosis of the nipple and areola (NHNA) is a rare skin condition of benign nature, which is characterized by wart-like, hyperkeratotic plaques involving the nipple, areola or both. It can be either unilateral or bilateral. The condition is usually asymptomatic; however, it causes cosmetic concern to the patients. Nevoid hyperkeratosis of the nipple is of unknown etiology; some subtypes are pregnancy-related and suggest a hormonal role. It may resemble in its appearance seborrheic keratosis and navus verrucosis, serious cancerous conditions must be excluded as well. This condition is observed in both males and females, with significantly higher prevalence among females. In this case report, we present a case of Nevoid hyperkeratosis of the nipple in a male patient, highlighting the clinical presentation, diagnosis and management options [1,2].

## Case presentation

**Figure 1:**
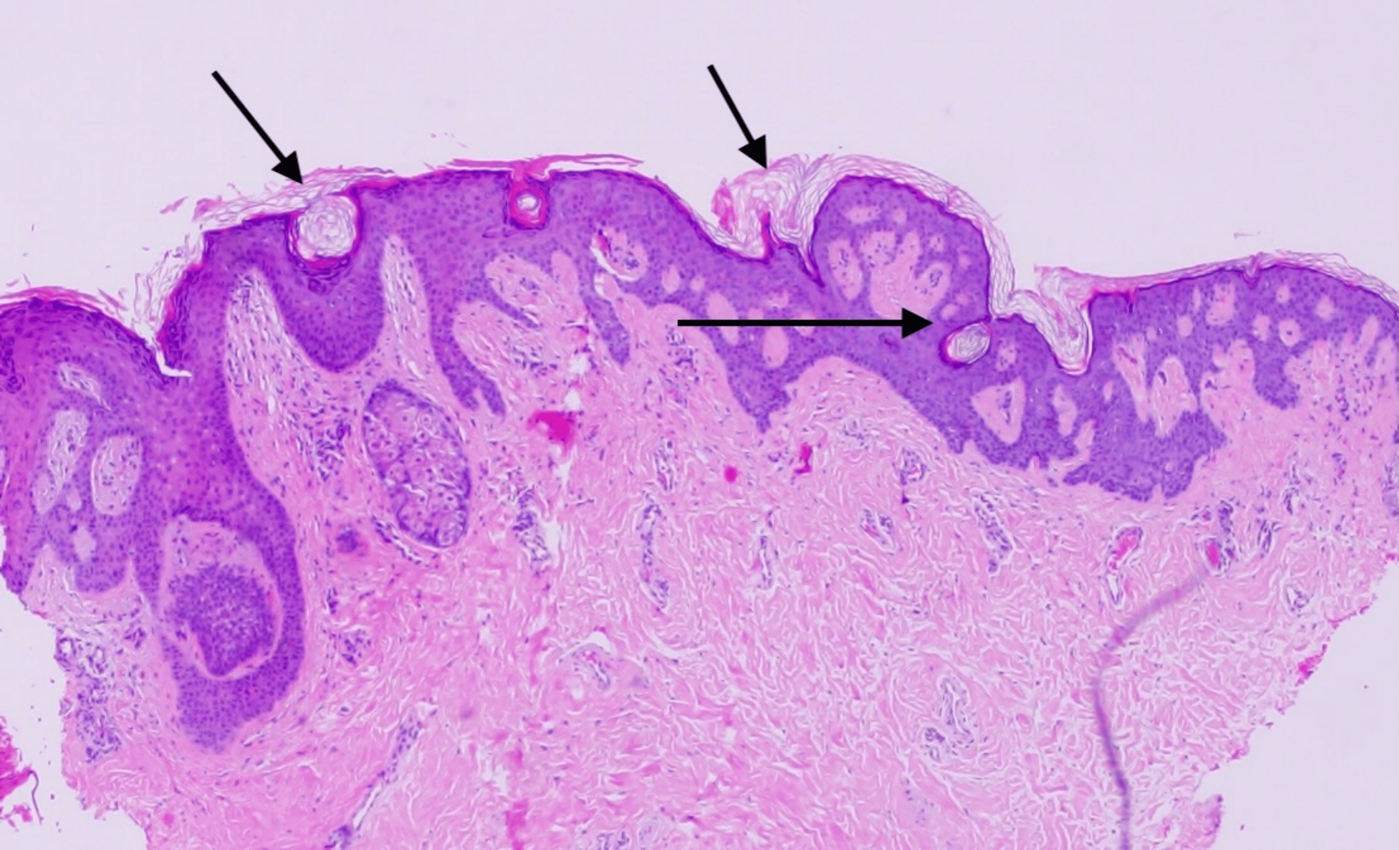
Hyperkeratosis, follicular plugging horn and pseudohorn cysts, acanthoses, papillomatosis, and elongated anastomosing rete ridges. Superficial perivascular inflammatory infiltrate forms of lymphohistocytic admixed and melanophages.

**Figure 2:**
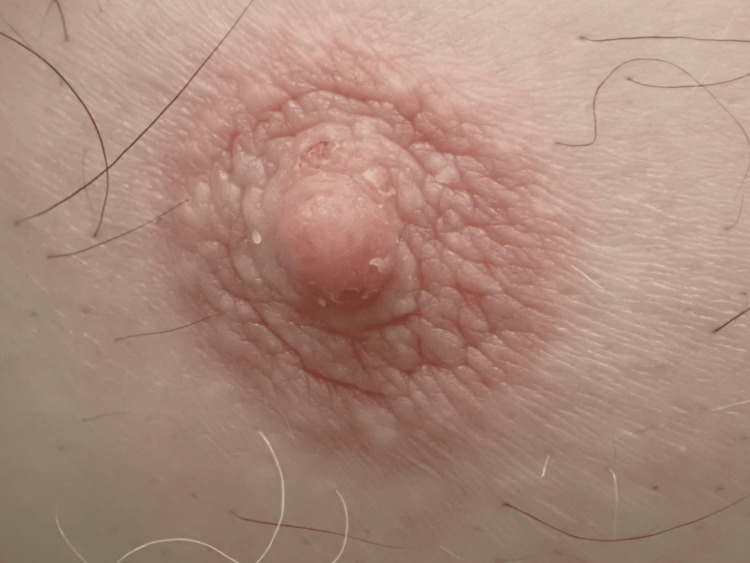
Hyperkeratotic, verrucous plaque with erosions and scaling over the right nipple and areola.

A 72-year-old Kuwaiti male patient presented to the dermatology clinic with a unilateral nipple lesion that has been causing him discomfort for the past six months. There was no associated history of nipple discharge, itching, bleeding or pain. On examination, the lesion was an erythematous, verrucous plaque with erosions and scaling over the right nipple and areola (Figure 1), no palpable lymph nodes and no similar lesions over the rest of the body. Bilateral breast examination was normal, and the patient has no history of using hormonal therapy. Paget’s disease and Paget's eczema were on the list of differential diagnoses. The patient was referred for a mammogram, which revealed no abnormal findings. Biopsy was done, and histopathology examination revealed marked thickening of the stratum corneum, papillomatous appearance, and minimal superficial dermal perivascular lymphocytic infiltrate, no evidence of cytologic atypia or dysplasia (Figure 2). Thus, a diagnosis of nevoid hyperkeratosis of the nipple and areola was made based on the clinical picture and biopsy findings. The patient was treated with topical steroids with a good response.

## Discussion

NHNA is a rare condition that is more common in females. The skin lesion classically manifests as hyperkeratotic plaques described as verrucous lesions. These lesions are typically painless, with no discharge or lymphadenopathy associated with them; however, some patients may experience pruritus, and women may report breastfeeding difficulty [1,2]. In our case, we had a presentation of the lesion that is unilateral lesion in a male patient.

Although the exact cause of NHNA remains unknown, Estrogen levels are thought to contribute to the pathogenesis, as the predominance of cases reported are in women of childbearing age; approximately 80% of cases reported in females of ages between 20-40 years old, and individuals receiving estrogen therapy for certain types of cancer. This supports the hypothesis of hormonal influence [2,3].

The most recent classification of nevoid hyperkeratosis of the nipple broadly classifies it into primary and secondary types. The primary form is idiopathic, usually bilateral and benign. Secondary type is associated with another dermatologic or systemic condition like epidermal nevus or seborrheic keratosis; it can be unilateral or bilateral, and further workup must be done to rule out underlying dermatosis or malignancies [2]. The current case happened to fall under the primary type.

The clinical appearance of the lesion may resemble other dermatological conditions like seborrheic keratosis, acanthosis nigricans, and more serious conditions like Paget’s disease and Paget's eczema. A proper diagnosis through skin biopsy is essential to exclude the potential serious conditions. For diagnosing NHNA, dermatoscopy can show structureless, garish or brown hyperkeratosis. Histopathological examination reveals marked hyperkeratosis, acanthosis and papillomatosis. The basal layer might show hyperpigmentation with a mild lymphocytic infiltrate of the superficial dermis. Most importantly, no atypical cells or pagetoid cells are identified, which helps exclude Paget's disease [4-6]. Our case matches the clinical and histopathological description of nevoid hyperkeratosis of nipple and areola.

When it comes to treating NHNA, both medical and surgical options are available to improve the appearance of the skin. Medical therapies include topical corticosteroids, retinoids and keratolytics like salicylic acid can be used. Multiple case reports showed excellent responses to cryotherapy, reporting complete disappearance of the lesion after six sessions. Other options, like carbon dioxide laser ablation, have a good cosmetic outcome. In some cases, surgical excision is used as an invasive method after all medical treatment fails, with no recurrence reported after two years [7-9]. This case was treated conservatively with topical steroids, with a good response.

## Conclusions

NHNA is a rare but benign condition; both males and females can be affected. Early recognition and exclusion of serious dermatological conditions is necessary through clinical evaluation and histopathology confirmation. Management can be conservative with topical steroids and keratolytics or non-conservative, including cryotherapy, laser, and surgical approaches. 
